# Supplementary material for: Improving the “real life” management of schizophrenia spectrum disorders by LAI antipsychotics: A one-year mirror-image retrospective study in community mental health services
Source: PLoS One. 2020 Mar 10;15(3):e0230051. doi: 10.1371/journal.pone.0230051 (PMC7064243; doi:10.1371/journal.pone.0230051)
Supplement: S1 Data — (ZIP) [file pone.0230051.s001.zip › SI data.docx]

*Data*

The clinical and electronic files of all patients attending five community mental health services of the Department of Mental Health of Bari (Italy) and receiving long-acting injectable antipsychotics (LAI) medications from July 2007 to June 2017 were analyzed.

Data Format (.cvs): ID;Gender;Age;illness duration;LAI gen;hosp. N. pre LAI;hosp. yes/no pre LAI;hosp. N. post LAI;hosp. yes/no post LAI;emerg. visits pre LAI;emerg. visits post LAI;planned visits pre LAI;planned visits post LAI
